# Topology for gaze analyses - Raw data segmentation

**DOI:** 10.16910/jemr.10.1.1

**Published:** 2017-03-13

**Authors:** Oliver Hein, Wolfgang Zangemeister

**Affiliations:** Neurological University Clinic Hamburg UKE, Germany

**Keywords:** gaze trajectory, event detection, topological data analysis (TDA), clustering, parameter-free classification, visual strategy, global scanpath, local scanpath

## Abstract

Recent years have witnessed a remarkable growth in the way mathematics, informatics, and computer science can process data. In disciplines such as machine learning, pattern recognition, computer vision, computational neurology, molecular biology, information retrieval, etc., many new methods have been developed to cope with the ever increasing amount and complexity of the data. These new methods offer interesting possibilities for processing, classifying and interpreting eye-tracking data. The present paper exemplifies the application of topological arguments to improve the evaluation of eye-tracking data. The task of classifying raw eye-tracking data into saccades and fixations, with a single, simple as well as intuitive argument, described as coherence of spacetime, is discussed, and the hierarchical ordering of the fixations into dwells is shown. The method, namely identification by topological characteristics (ITop), is parameter-free and needs no pre-processing and post-processing of the raw data. The general and robust topological argument is easy to expand into complex settings of higher visual tasks, making it possible to identify visual strategies.

## Introduction

Gaze trajectories can tell us many interesting things about human nature, including attention, memory, consciousness, etc., with important applications [55, 36, 168, 131] as well as facilitating the diagnosis and helping to understand the mechanisms of diseases [94, 111, 28]. Normally, viewing behavior is studied with simple paradigms to keep the complexity of natural viewing situations as low as possible, e.g., in a search paradigm, a person looks at a computer screen with a simple static geometric configuration under well defined optical constraints, i.e., constant illumination, head immobilized by a chin rest or bite bar, no distractors, etc.

The task of analyzing, classifying, and interpreting gaze trajectories for realistic situations proves to be much more difficult because of the many different factors influencing the steering of the eyes. The usual scientific approach is to break down real world complexity into easy to define and control partial modules, and then to try to reassemble reality from these simple modules. This has also been done for gaze trajectories. The task of analyzing the gaze trajectory data can roughly be split into two subtasks: the low level description of the noisy raw data that are produced from the gaze tracker, and the high level description of the data in combination with the viewing task and the cognitive processes. The first subtask could be regarded as the mathematical modeling of high frequency timeseries, given that modern gaze trackers can sample eye position and orientation at 2000 Hz or even more [4].

The careful choice of the data model and data representation is the basis for all of the following analyses. Only a model capable of incorporating the many subtleties of the gaze trajectory is able to support the complex questions which appear in the context of modeling the looking task in relation to the assumed cognitive processes^1^. 1 Of course, a more complex model is harder to implement and interpret. There is a permanent balancing between data load, explanatory potential, and model complexity.

## Splitting trajectory data into events

In this section a general outline of splitting raw eye-tracking data into meaningful events is given. At present, the most important segmentation of the data is the dichotomous splitting into fixations and saccades. Although this is a long standing approach, up to now no definite algorithm for the splitting exists. The reasons are discussed.

### The basic oculomotor events

The eyes’ scanning of the surrounding is done in a sequential manner, since the movement of the eyes, seen as a mechanical system, is limited to sequential movements. It has to be remarked that, in many aspects, this is not true for the information extraction and processing of the visual data within the brain, which can process information in parallel [157, 160]. It is well known that a detailed analysis can only be done for a very small part of the visual scene, approximately 1 up to 5 degrees of visual angle [27, 37]. This is the part of the scene which is projected onto the fovea, the region of the retina with the highest concentration of cone cells. To capture the whole scene, the eyes have to switch swiftly to other regions within the scene, which is done via saccades, i.e., very fast movements [47, 88]. In fact, saccades are operationally defined by velocity, acceleration, and amplitude criteria. Saccades exhibit a clear characteristic, which is relatively stable across subjects [95]. Quantitatively this relationship is expressed in the main sequence [11, 10, 9, 20]. Speed is crucial, because the brain has to integrate many parts of the whole scene into one consistent and stable internal representation of our surrounding world, and because of the fact that the observer has decreased sensitivity while the eyes are moving fast, a phenomenon called saccadic suppression [103, 95]. Information gathering works by swiftly scanning the scene and minimizing the timespan of decreased sensitivity. This fact makes a bipartition of the gaze trajectory data desirable.

The gaze trajectory is broken down into two general subsegments, fixations and saccades. Saccades allow the gaze to change between parts of the scene, while fixations are intended for analyzing parts of the scene. Saccades are the segments of the trajectory where eyes are moving fast and in a preprogrammed, directed manner, whereas in a fixation eyes are moving slowly and in a random-like fashion [129]. The two modes of movement are displayed alternatively and exclusively. Fixations may then be defined as the part between the saccades or vice-versa. This is a sensible and convenient assumption, but also a major simplification. It is well known that fixations can contain microsaccades as subitems [100, 129, 42], mixing the two assumed modes of movement.

These two different movement characteristics can be operationalized. The bipartite classification of gaze points in saccade points and fixation points is normally achieved through a combination of space and time characteristics, i.e., for a fixation, the dispersion of the gaze points on the display combined with the duration of a cluster of gaze points in time; for a saccade, it is the velocity, acceleration, and amplitude of the movement. The exact determination of the parameters and the algorithmic implementation has a long history and many parameterizations exist^2^.

The classification of eye movements into fixations and saccades is by no means straightforward. One always has to bear in mind that the dichotomic splitting of the data follows our desire for simple and parsimonious models,3 it is not Nature’s design^3^. It has to be noted that the eye has a much broader repertoire of movements [97]. “Patterns” of eye movements other than fixations and saccades occur in real data, e.g., vestibular and vergence eye movements, dynamic over-/undershooting, microsaccades, drift, tremor, etc. This becomes even more complex when viewing dynamic scenes as opposed to still images [28]. Because of the moving content, the eyes have to follow the infocus part of the scene. The concept of a fixation as being localized in a small subregion of a still image is no longer valid and has to be replaced by the concept of smooth pursuit [18]. As of now the most important event types are fixations, saccades and smooth pursuit. More recently post-saccadic oscillations (PSO) have come into focus [116, 3]. Zemblys, Niehorster, Komogortsev, and Holmqvist [186] estimate 15-25 events that, as of now, have been described in the psychological and neurological eye-movement literature.

As common for biological systems, all movements exhibit a normal physiological variability [146, 166]. Different application regimes also show different characteristics, e.g., normal reading is different from reading a drifting text [165] as it is now common when reading, or even browsing, texts on mobile devices (swiping the text). Furthermore, gaze tracking data can be interrupted by blinks. Blinks interrupt the flow of gaze tracking data, while the eye is still moving consistently. Though coupled [67], blinks are considered noise.

Even if all possible events were known and clearly defined, the algorithmic processing would introduce a bias into the results. There are many reasons for this finding. One reason lies in the different sensitivities to noise and filter effects [68, 159], e.g., numerical differentiation is an operation with notorious “bad behavior”. Furthermore, the filters used for preprocessing also call for parameters and introduce a bias into the data.

### Higher level use for oculomotor events

Another motivation for the development of more and more sophisticated algorithms is the growing – one might say exploding – applicability of eye tracking devices. In the past eye tracking was restricted to scientific uses and the tasks people were performing were relatively low in complexity, e.g., a simple search task. Nowadays, with the increase of performance in eye-tracking hardware and computing power, the tasks under investigation have become more and more complex, producing a wealth of data.

Recent years especially have shown a growing interest in the investigation of complex dynamic settings. In these settings the viewing subject is no longer looking at a static image from a (head-)fixed position. In the extreme, the subject is moving freely and interacting with its environment, like playing table tennis or driving a car [90, 89, 91, 92]. Driven by industrial applications such as market research, dynamic scenes are playing a more and more important role. These can be watching TV and movies [52, 22, 34], video clips [26, 15, 161] or interactively playing a video game [121, 152]. Another application is the assessment of the driving ability in diseases like glaucoma [28] or Parkinson’s Disease [23], where patients view hazardous situations in a car driving context. The system calibration can be automated, allowing the collection of data for many subjects. As an example, the eye movements of 5,638 subjects have successfully been recorded while they viewed digitized images of paintings from the National Gallery collection in the course of the millennium exhibition [181, 182, 180]. It is apparent that such data sets can not be evaluated manually. A recent application is online tracking of eye movements for integration in gaze contingent applications, e.g., driving assistance, virtual reality, gaming, etc. Here the online tracking produces a continuous stream of highly noisy data, and the system has to extract the relevant events in real time and has to infer the users’ intents to adjust itself to their needs.

These more complex settings and large sample sizes are not only a challenge for the hard- and software, but also require a rethinking of the concepts being used to interpret the data, especially when it comes to the theoretical possibility of inferring people’s intent from their eye movements [59, 21, 54].

In summary, the analysis of eye tracking data can be organized in a hierarchy spanning different scales, going from low level segmentation ascending to higher levels, relevant for the physiological and psychological interpretation. Topmost is the comparison and analysis of different eye movement patterns within and between groups of people, as is relevant for the inference of underlying physiological and cognitive processes, which forms the basis for important eye tracking applications, see Table 1 Highlighted in light gray background is the first level aggregation into basic events. Highlighted in dark gray is the second level aggregation for higher use, i.e., sets of sequential fixations in a confined part of the viewing area [135]^4^.

**Table 1:**
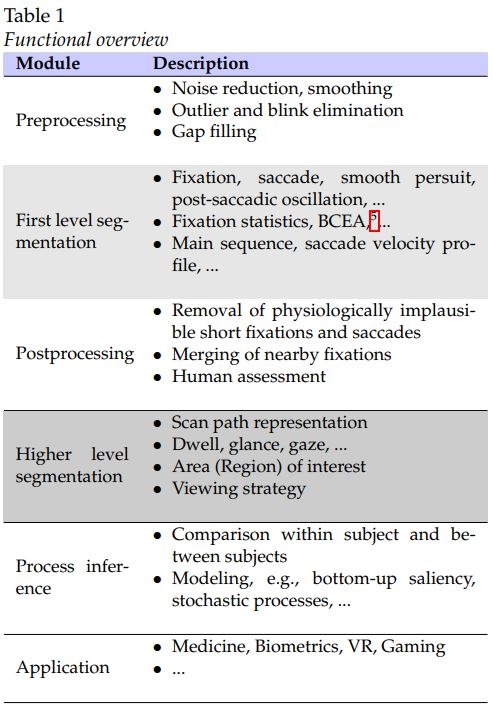
Functional overviewe

### The problem of defining a fixation

For most areas of inquiry this level of information in the raw data is not necessary. It is sufficient to reduce the gaze-points into oculomotor events, i.e., into the fixations and saccades forming the scanpath. Here scanpath 6 means any higher level time ordered representation of the raw data which form the physical gaze trajectory. The fixations can further be attributed to regions of interest (RoI), each RoI representing a larger part of the scene with interesting content for the viewing subject.

While intuitively easy to grasp, it is by no means obvious how to explicitly define these concepts and make them available for numerical calculations [3]. Very often only basic saccade and fixation identification algorithms are part of the eye-tracking system at delivery ([156], leaving the higher splitting up to the user. This is desirable in the academic setting, but not in the industrial setting, where time efficient analysis has to be conducted, e.g., in marketing research [127] or in usability evaluation [51]. Most commercial implementations incorporate dispersion threshold methods, e.g., ASL [7] or velocity threshold methods, e.g., seeingmachines [141]; Olsen [117]; Tobii [158]. Some offer the user flexibility in choosing the thresholds, while others mask the complexity from the user by assuming a sort of lowest common denominator for the thresholds in different application domains, although it is known that parameters can vary between different tasks, e.g., the mean fixation duration amounts to 225 ms on silent reading, 275 ms on visual search, and 400 ms on hand-eye coordination [125]. To account for these variations, some implementations have 10 parameters to adjust [126], requiring a good understanding of the theory of gaze trajectories.

It is well known that the parametrization of the algorithm can substantially affect the results, but there is no rule which algorithm and which parametrization to employ in a given experimental setting [149, 116, 177]. A comparison of the different algorithms and the bias which can result under different parameterizations is given in Shic et al. [145]; Špakov [176]; Andersson et al. [3]. For instance, post-saccadic oscillations (PSOs), i.e., wobbling over/under-shootings, are usually not explicitly mentioned, but form a normal part of eye movements. The PSOs are attributed to fixations or saccades, influencing the overall statistics of the measurement [116, 3]. The algorithms to implement the classification are therefore different and researchers aim to improve and extend the algorithms constantly [166, 176, 83, 108, 96, 175, 177, 165, 31, 3, 63, 186].

Many researchers agree that a normative definition and protocol is desirable but at present far from becoming reality [77, 81, 116, 3]. As Karsh and Breitenbach [77] stated rightly:

The problem of defining a fixation is one that perhaps deserves more recognition than it had in the past. Generally speaking, the more complex the system the more complex the task of definition will be. ... Once these needs are recognized and implemented, comparison between studies take on considerably more meaning.

### Topological approach to the problem

Up until now, no single algorithm has been able to cover all the various aspects in eye tracking data [3]. The aim here is to show that there exists a strikingly simple argument for demarcating the different components of the gaze trajectory in a normative way. From well-known approaches a data representation is derived, which forms the basis for a consistent analysis scheme to cover the basic aggregation steps, see gray parts of Table 1. The argument for the segmentation is a topological one and is by its very nature global and scale-invariant. It is the mathematical formulation that a fixation is a coherent part in space and time. The meaning of “coherent in space and time” will be clarified in the next sections. The argument needs no thresholds or calibration and is independent of any experimental setting or paradigm. The delineation of the gaze trajectory is unambiguously reproducible.

## Overview of existing approaches

This section presents an overview of different approaches to event detection. From these, a common argument is isolated, the coherence of sample data in space and time, which in turn forms the basis for the new algorithm.

### Taxonomy of algorithms

At present, we see a wide variety of different methods being used to extract the main oculumotor events from raw eye tracking data [64]. Each approach to the data highlights at least one prominent and distinguishing feature of the main oculomotor events in the trajectory data and makes use of specialized algorithms to filter/detect these features against the noisy background. Noise is to be understood as being the part of the measurement which is not relevant for the investigation, e.g., micro saccades can be considered noise in one study, but be of central interest in another setting. In its narrow sense noise is the random part inherent in any measurement. There is a common logic to all these approaches, from which a data representation and global topological argument can be derived. To better understand the topological approach, algorithms currently in use are systematized in a taxonomy. The taxonomy was first introduced in Salvucci and Goldberg [134]. This classification has often been repeated and adapted in the literature [81, 80, 83, 136, 3]. Here, as in Salvucci and Goldberg [134], the classification is based on the role of time and space as well as algorithms used to evaluate raw data. Broadly speaking, there are two different approaches to the data, which differ in complexity.

The algorithmically simplest approach is based on thresholds for saccades and fixations. In the case of saccades these are thresholds for velocity (I-VT: identification by velocity threshold), acceleration, and even jerk, very often calculated as the discrete numerical space-time n-point difference approximations to the continuous differentials. E.g., a saccade is detected whenever the eye’s angular velocity is greater than 30 deg/s [122, 150, 44, 48, 120]. These algorithms are called “saccade pickers” [76].

The second group targets the space dispersion (I-DT: identification by dispersion (position-variance) threshold) or space-time dispersion (I-DDT: identification by dispersion and duration thresholds), i.e., when a consecutive series of gaze points occur near each other in display space, they are considered part of a fixation. E.g., in a reading context, a fixation lasts between 200 and 300 msec and a saccade spans approximately seven character spaces [125]. Gaze points consistent with this are aggregated and assumed to form a single fixation. These algorithms are called “fixation pickers”. Most algorithms use simple thresholds to cluster data into saccades and fixations, which in practice need to be optimized. A fixed parameter approach may perform well on a specific record but is very often too imprecise and error-prone when applied to different records 7. In order to improve results, researchers adapt the threshold in a dynamic way [41, 116], or combine criteria, e.g., a saccade is detected when the angular velocity is higher than 30 deg/s, the angular acceleration exceeds 8000 deg/s2, the deflection in eye position is of at least 0.1 deg, and a minimum duration of 4 ms is exceeded [154, 46, 147, 148]. Note that dispersion thresholds can be inversely defined for saccades, i.e., in relations to a fixation, a saccade is over-dispersed, i.e., it has a minimum jumping distance. This is essential when delineating micro saccades from saccades.

Parameters are often chosen subject to individual judgment or even rather arbitrarily [70]. Even after using more criteria, human post-processing is required [177], and means to reduce the human interaction are being sought [32].

A higher sampling rate of the eye-tracker will give better approximations of velocity and acceleration, but the devices are more expensive and demand higher restrictions for the tested subjects, e.g., a chin rest, etc. It is remarkable that functional relationships like the main sequence [11] are rarely employed, considering that they give good guidance for setting parameter thresholds [68]; a recent exception is Liston et al. [96].

All these approaches are purely operational, call for experience, and are driven by technical as well as programming restrictions. More complex algorithms are of course harder to code and often suffer from performance issues. The simple velocity and dispersion based classifiers are exemplified in Table 2 (citations contain an explicit exposure of algorithm).

**Table 2:** 

saccade pickers	
d/dt velocity threshold I-VT	fix (Stampe, 1993[150])
	adaptive (Nyström and Holmqvist, 2010[116])
d^2^/dt^2^ acceleration threshold I-AT	fix (Behrens and Weiss, 1992[13]; Behrens, MacKeben, and Schröder-Preikschat, 2010[12])
d^3^/dt^3^v jerk threshold I-JT	fix (Wyatt, 1998[183]), (Matsuoka and Harato, 1983[104], in Japanese)
fixation pickers	
dispersion threshold I-DT	fix (Mason, 1976 [[Bibr b102]]; Kliegl and Olson, 1981[79])
dispersion and duration thresholds I-DDT	fix (Widdel, 1984[179]; Nodine, Kundel, Toto, and Krupinski, 1992[112]; Manor and Gordon, 2003[99]; Krassanakis, Filippakopoulou, and Nakos, 2014[85])

A considerable advantage of these approaches is that thresholds are easy to understand, interpret, and implement. The values for thresholds depend on research domain, e.g., the space-time dispersion values in I-DDT are different in reading and in visual search. Fixation times are domain specific, i.e., the duration of a typical fixation in reading is different to fixation times in visual search, etc. [125]. Hand-tuning is often requisite to get good results and is based on heuristics.

### Range of advanced methods

The more sophisticated algorithms use ramified versions of the basic velocity/dispersion features taken from signal processing, statistics, Kalman filtering, Bayesian state estimation, clustering, pattern classifier algorithms, and machine learning.

These are taken from other disciplines like

Signal processing – Finite impulse response filter [159] – Cumulative sum (CUSUM) [118, 158, 58]

Statistics – F-test and correlation [173, 174, 172] – Gap-statistics [108]

Stochastic processes and time series analysis – Auto-regressive processes and wavelet analysis [35]

Bayesian approaches – Hidden Markov model [133, 130] – Kalman filter [137, 84] – Bayesian mixture model [153, 78] – Particle filter [31]

Data clustering – k-means clustering [124] – Projection clustering [163] – Mean shift clustering [135] – Mean shift clustering and entropy [171] – Two-means clustering [63]

Machine learning – Random forest classifier [186] – Neural networks [66, 1]

Graph theory – Minimum spanning tree [50, 128]

Fuzzy-set methods – [6, 30]

Shape features^8^. – Single feature (simple) [74], [15] – Multiple features (complex) [175] – Mathematical morphology [98]

Speech recognition – Mel-frequency cepstral analysis [29]

Template matching – Velocity-Duration template [96]

Dynamic system analysis – Time-delay reconstruction [142, 143]

As of now threshold based methods are common standard. Probabilistic methods are promising candidates inasmuch as they offer the possibility to implement an online learning algorithm to adjust to changing viewing behavior. Very recent candidates for event classification are neural networks [66, 1], random forests [186] or machine learning in general [185].

## Topological data analysis

A relative recent field of data analysis is topological data analysis (TDA). In this section, a topological approach to the data is given. To this end, the notion of different spaces, projections and metrics for the trajectory is introduced. The idea of trajectory spacetime coherence coherence is given a precise meaning in topological terms, i.e., “no holes in trajectory spacetime”, a strikingly simple topological argument for the separation of the sample data. An intuition and first use for the argument is given by the visual assessment of the trajectory spacetime, showing the coarse/fine (global/local) structure of a scanpath.

### Configuration in physical space

The crucial aspect for partitioning the data is the representation of space and time. Space is here understood as the three-dimensional physical space, called world space, which contains as objects the viewer, items viewed, and tracking equipment. Essentially, the viewer’s head and eyes have position (location) and orientation, together called pose, in world space. In the case of the eyes, very often only the direction is determined. The starting point for analysis is the set of raw data from the gaze tracker. The logging of continuous movement of head and eyes consists of the discretely sampled position and orientation of head and eyes in three-dimensional space at equidistant moments in time during the timespan of the experiment.

If it were the intention only to detect fixations or saccades, it would be sufficient to analyze the movement of the eyes in head space. In the context of, e.g., cognitive studies, position and orientation of head and eyes is not interesting in itself; of interest are the visual field, the objects within the visual field and the distribution of allocated attention within the viewer’s internal representation of the visual field, “the objects looked at”. Because of this, the motion of the visual field in world space will be modeled.

The visual field encompasses the part of the environment which is in principle accessible for gathering optical information. It is well known in visual optics that the way of light from an object onto the retina is a multistage process which depends on the optical conditions in world space as well as the geometry and refractive power of the different parts of the individual eye [5, 107]. Taken together, this is a complex setting to analyze.

In order to cope with the complexity, several assumptions and simplifications have to be made in the course of modeling. The visual field is not directly accessible to the eye tracker. The eye tracker can only measure related signals. These signals are linked by calibration to the point of regard. E.g., in video based head-eye tracking, camera(s) take pictures of the head and eyes of a subject. The individual images are processed to identify predefined external features of the head and the eyes, e.g., the corners of the mouth and the eyes, the pupil, and glints from light emitting diodes on the light reflecting surfaces of the eyes. From the relative position of these features in image space(s) and the calibration, the gaze 9 can be determined.

The visual field for one eye is approximated as a right circular cone of one sheet with the gaze-ray as its axis, the center of the entrance pupil as its apex, and with a varying aperture, neglecting any asymmetry of the visual field. For foveated objects the cone angle of a bundle of rays that come to a focus is very small, approximately 0.5 degrees. In the limit of 0.0 degrees only a ray remains, which is convenient for calculations. One calculates the point of intersection of the gaze-ray (starting from the center of the entrance pupil) with an object in world space, and not the projection of the content of the gaze cone onto the retina. Very often one does not work with the gaze-rays of the two eyes separately but instead with only one of the two (the dominant eye); alternatively, the two gaze-rays are combined into a single gaze-ray, i.e., a mean gaze-ray known as “cyclops view” [39]. In addition, very often the head is fixed to prevent head movements at the cost of a somewhat nonphysiological setting.

To describe the geometric and topological approach to the data in detail, we will choose the situation where a subject is looking at a screen presenting a visual task (which is a common experimental setting). The point of regard (PoR) is the location toward which the eyes are pointed at a moment in time, i.e., the point of intersection of the (mean) gaze-ray with the screen. Please note that the topological method can work just as well in a three-dimensional setting, e.g., navigating in outdoor scenes. The 3D case is of recent interest for orientation in real and virtual space. For the sake of clarity of explanation, we will now discuss a typical two dimensional setting.

### Coherence in space and time

The rationale behind the intended clustering is that trajectory points which have a certain coherence in space and time should be grouped together. The question is how to define and express spacetime coherence for trajectory points. The argumentation starts with the continuous gaze trajectory tr. The gaze trajectory consists of the time-ordered points of intersection Pts of the mean gaze-ray with the screen or screen space and#x03A3;, within the timespan ts of the experiment. In mathematical abstraction:

**Figure:**
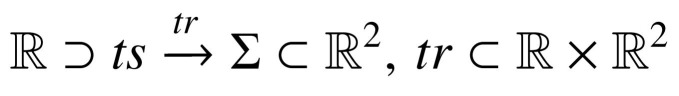


**Figure 1:**
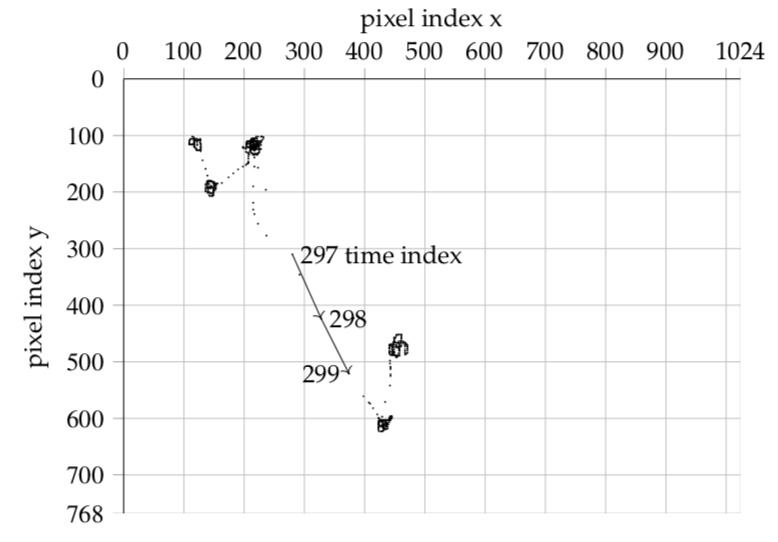
Trajectory in screen space

The terminology and notation is not a mathematical pedantism. In the following, different spaces will be introduced and it is essential not to lose track of one’s current conceptual location. It is important to note that the unparametrized Ps form a multiset because the gazeray can visit the same screen point at many time points (within a fixation and recurrently). Contrary to screen points, a time point, representing an instant or moment in the flow of time, can be visited or passed only once. In practical terms we only have a finite number of discrete data, i.e., the protocol pr of sampled tr. The pr results from a discretization of continuous space and time. The screen consists of a finite number of square pixels all with equal side length ∆x = ∆y = constant, the constituting discrete elements of screen space Σ' = {Px;y : x 2 ް 1 1023} y 2 {0, 1, ..., 767}}, here XGA resolution is assumed, and the tracker takes pictures at moments in time with a constant sampling rate (time points or moments) *ts'* = *{M*_i_:Iϵ{O,1,..,N-1} therefore *pr = {P_M0_ , P_M1_ , P_M2_ ,..., P_Mn_*}. Time is considered to be an ordering parameter, and because of the constant sampling rate, only time index is noted pr = (P_0_, P_1_, P_2_, ..., P_n_) with the ordering parameter *i* ϵ ℕ_0_. It is important to note that the points of intersection alone do not carry any time information. If we want to convey the information about time ordering, we must label points, i.e., show the index. Graphically we can also show a polyline with the line segments sensed, i.e., showing an arrowhead, see Figure 1

The crucial step for the following is to take a different position with regard to the subject, the combinatorial view. In analogy to space dispersion algorithms, the spatial distance of two points is taken, but this time not only for consecutive points in time but all possible 2- point combinations over time. This could be regarded as taking the maximal window size in the dispersion algorithms. This way one obtains the time indexed matrix D of all combinatorial 2-point distances for the trajectory space. D serves as the basis for further evaluation. The representation as a time indexed matrix of combinatorial 2-point distances makes the trajectory independent of Euclidean motions because distances are the invariants of Euclidean geometry. The property of being independent of Euclidean motions is especially desirable when comparing scanpaths [71]. At first sight this approach may seem to resemble a superfluous brute force dispersion approach. The advantage of such an approach will be clear from the subsequent sections.

First, we can make the spatio-temporal relationship of the Pis directly visible with an imaging technique. To this end, we convert, for all time ordered pairs of trajectory points (Pi; Pj); the screen space distance values d_i,j_ into gray values of a picture, img(D); of size |pr|X|pr|. E.g., when the gaze tracker takes 633 samples one obtains an image measuring 633 by 633 pixels^10^.

**Figure 2:**
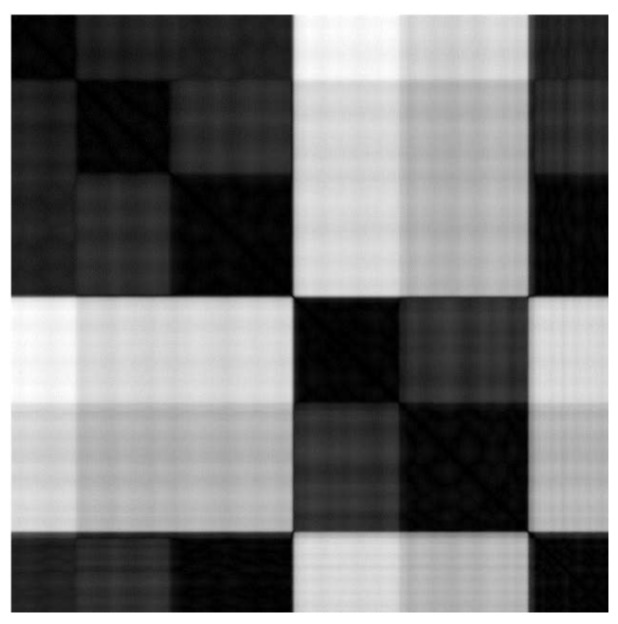
Image of time indexed matrix of 2-point combinatorial distances img(D)

**Figure 3:**
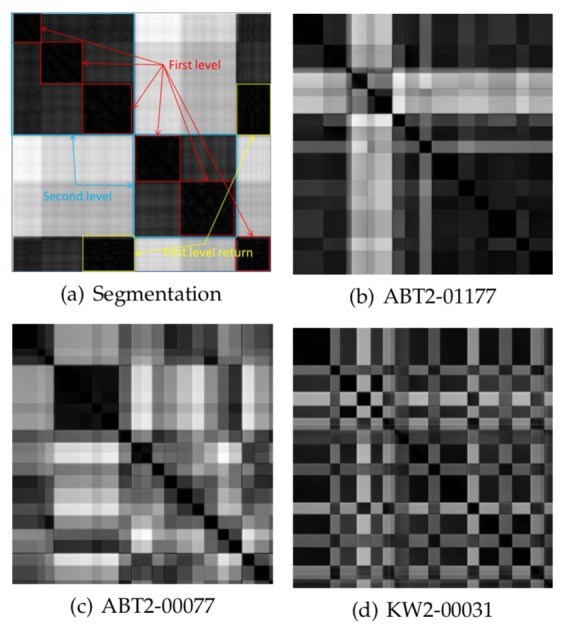
Hierarchy of sample clusters, first level are fixations, second level are clusters of fixations, rectangles of the first off-diagonal represent saccades

In the first line Figure 2 should seem suggestive. For the visual system of the human observer, the square block structure of img(D) along the diagonal is easy to identify. The squares along the diagonal represent the fixations. While fixations are spatially confined, their sample distances are short and their gray level is near black. The duration of a fixation is the diagonal (side) length of the square. The first off-diagonal rectangles represent the saccades between successive fixations. Spatially wider saccadic jumps are brighter and shorter jumps are darker. The building blocks form a hierarchy. First level squares are the fixations, second level squares are clusters of fixations, and so on, see fig. 3 (a). The hierarchy of squares along the diagonal is the visual representation for the trajectory (screen)spacetime coherence over different time spans, i.e., the scaling property in time. The scale runs from the base-scale, set by the sampling rate of the tracker, into its first physiological scale, i.e., the time-scale in a single fixation, showing, e.g., tremor, drift, and microsaccades, into the time-scale of several fixations within a dwell, viewing interesting regions, and finally into the time-scale of shifts in interest, changing the viewing behavior.

### Visual assessment of trajectory spacetime

The higher level splitting of the viewing behavior in space and time is a much debated subject [170]. The rationale comes under various names in different contexts. At its base, there is a dichotomy in terms of global/local [57, 106, 56], coarse/fine [119, 49], ambient/focal [62], where/what [144], examining/noticing [178], which is backed by anatomical findings, i.e., the concept of a ventral and dorsal pathway for visual information processing [162, 144].

If this dichotomous splitting is right, it would be sensible to find a corresponding splitting in the output of visual processing, i.e., in the spatio-temporal pattern of fixations and saccades. Here, the visual assessment of tendency of the spacetime representation will proove helpful. As an example, in Figure 3, three scanpaths from the publicly available database DOVES [167] are shown. DOVES contains the scanpaths of 29 human observers as they viewed 101 natural images [169]. Studying human viewing behavior while viewing pictures and images is a common subject in vision research. Since the seminal work of Buswell [24], one often repeated general statement is that people tend to make spatially widely scattered short fixations early, transitioning to periods of spatially more confined longer fixations as viewing time increased [8]. This behavior is exhibited in fig. 3 (b). Here, observer CMG2 looks at stimulus img01019. Visible are three major second level blocks. The classical interpretation would be that the second block, with its more variable structure, reflects the global examining phase, while the following more homogeneous block reflects the noticing phase. The first block at the beginning represents the well known central fixation bias in scene viewing [155, 17].

Interestingly, the database contains also good examples for the inverse behavior, e.g., observer ABT2 looking at image img00077, see fig. 3 (c). Here the spatiotemporal pattern could be interpreted as: first the central fixation bias, second a local noticing, and only then a global scanning. This behavior is not uncommon, as Follet, Le Meur, and Baccino [45] have noted.

These are only two examples from the database DOVES, which contains approximately 3000 scanpaths. The visual inspection makes it possible to get a quick overview of the spatio-temporal patterns for many scanpaths and to get an intuitive understanding of prevailing pattern classes. Scanning DOVES visually shows that a significant portion of the scanpaths exhibit a spatio-temporal pattern which does not fit into the classical coarse-fine structure, e.g., subject KW2 looking at img00031 in fig. 3 (d). Of course, the examples are cursory and it is not our intention at this stage to discuss image scanning behavior. The purpose of the examples is twofold: firstly, to show that by a visual assessment of img(D)s, one can reach a good intuitive understanding of spatio-temporal patterns and regularities in scanpaths. The human visual system is an excellent pattern detector, a resource for investigations that should be utilized, notwithstanding the fact that a statistical examination of the data and the statistical test of hypotheses must confirm “seen” patterns. The search for simple scanpath patterns is a common task for many research questions [105].

Secondly, that the time course of the scanpaths is an important factor, especially when discussed in the context of top-down strategies versus bottom-up saliency. A good quantitative model should replicate the empirical observed spatio-temporal pattern classes, reflecting the order of transits between different scanning regimes and their internal substructure. The whole pattern shows a global statistics as well as substatistics in the different regimes. When modeling scanpaths, very often scanpath data are aggregated into simple feature vectors containing summary statistics as features, i.e., mean number of fixations, mean fixation duration, mean saccadic amplitude, etc. A model is considered good if it can replicate the empirical summary statistics. This neglects any time course and hierarchy in the patterns.

The next step will be to exploit the representation as a time indexed matrix of all combinatorial 2-point distances as a precise instrument of trajectory segmentation and interpretation.

### Homology for spacetime coherence

At this stage, the human visual system has still been serving as pattern detector. The goal is to extract the interesting part of the information about the hierarchical spatio-temporal configuration of fixations, clusters of fixations and returns from the distance representation, and to do so on an automated basis, without any user defined parametrization, in a robust way. The question is how to express and implement this coherence algorithmically. The task will be accomplished in three steps.Step 1(see also Figure 4

**Step 1:**
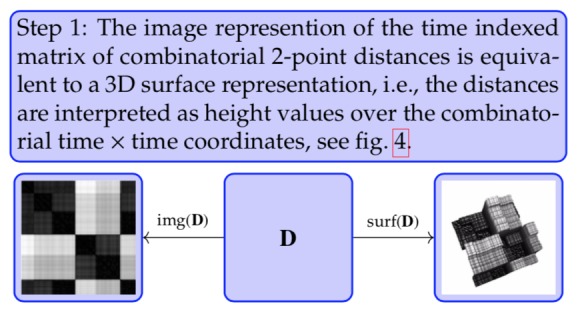


**Figure 04:**
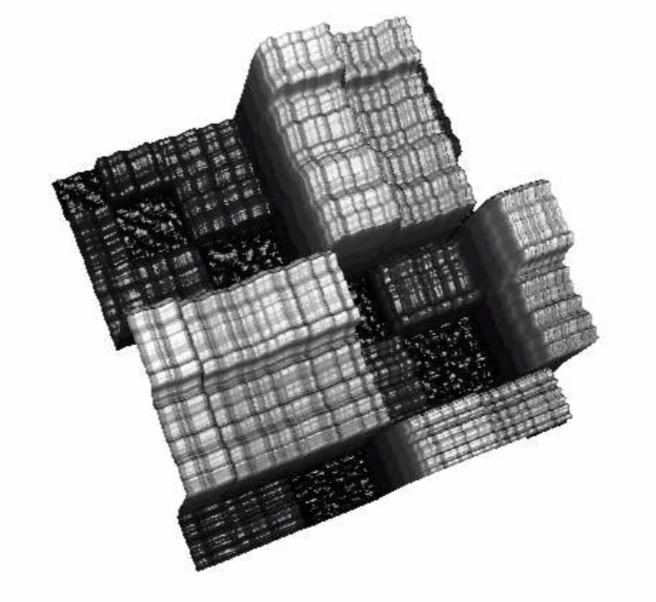
Surface plot of time indexed matrix of combinatorial 2-point distances

Clearly visible in the surface plot representation are rectangular columns with a small on-top variation. The small variation in blocks is considered noise. In the image view it could be regarded as a kind of texture. For a better intuitive understanding of the topological approach consider the 3D surface plot as kind of a landscape which is progressively flooded. Coherent are parts of the landscape which are below a certain sea level and form an area like a lake, without internal islands. Lying under or lying above sea level is filtering the level values according to a threshold. This is done in the next Step.

**Step 2:**
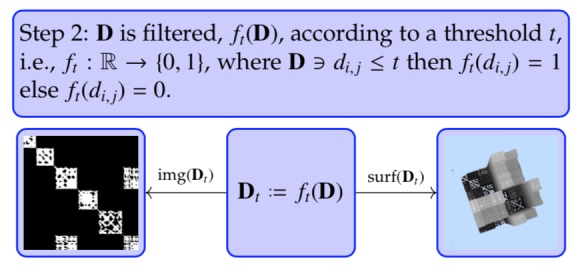


**Figure 5:**
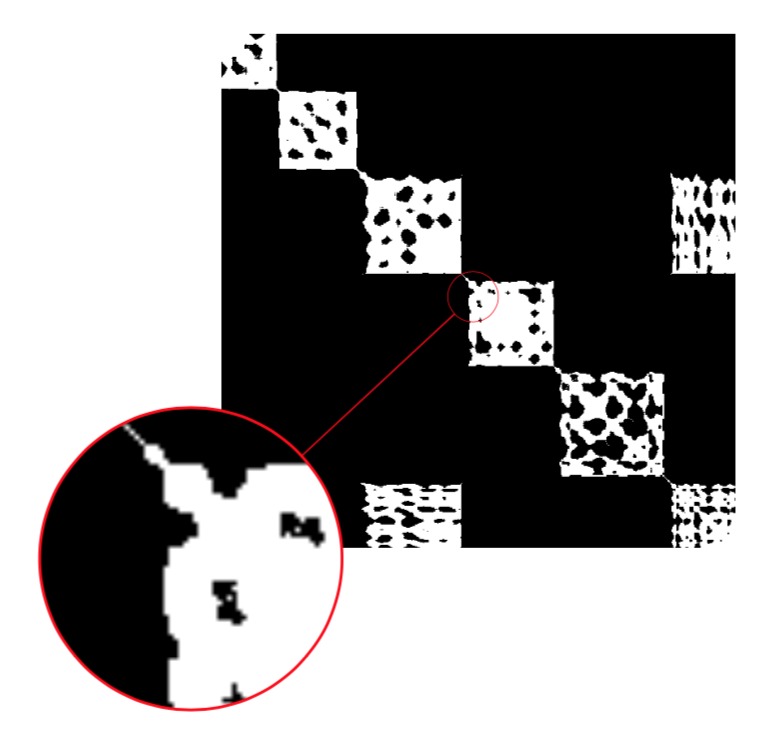
Filtered time indexed matrix of combinatorial 2-point distances. Magnification shows small components.

Notice the punctuated block structure in the image representation img( ft(D)), see Figure 5. While the overall square block structure along the diagonal and the off-diagonal rectangle block structure is still visible, the holes are representing the incoherence or noise. The incoherence is eliminated by closing the holes, i.e., raising the threshold (Step 3.

**Step 3:**
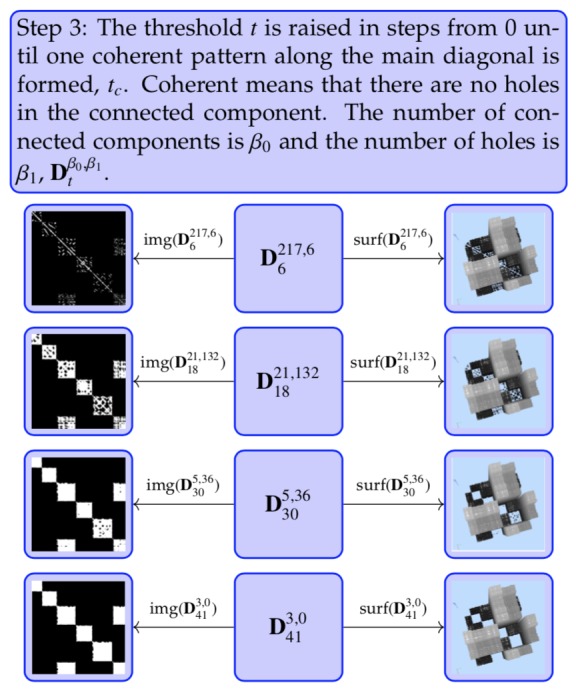


The coherent white part along the diagonal in the image representation is the partition of the data that we have been seeking.

It should be stated explicitly that the parameter tc for separation is not preassigned. The definition for separation is the coherent structure/pattern of trajectory spacetime. The distance threshold is increased until coherence is reached. This is done individually for every trajectory. The pattern is global for the trajectory and does not depend on local specifics. It is important to note that a more detailed analysis within each block will separate the noise into physiological noise (tremor, drift, micro saccades, etc.) and instrument noise. In the supplementary document this approach can be interactively investigated.

All this is easy to understand for human intuition, but needs a formal mathematical theory along with an algorithm and efficient computer implementation. Generally speaking, there exist three methods to tackle the problem. The first is the obvious way, i.e., a human observer varies the “sea level”. Human evaluation especially of noisy data is common practice in eye tracking data analysis ([132]. The second way is using a simple “brute force” image analysis algorithm. The third, more elegant, way is to use algebraic topology in the form of homology. Homology tells us about the connectivity and number of holes in a space, in our representation the “islands and lakes” created while flooding the space. Counting the number of connected components and the number of holes is calculating the first two Betti numbers, *β_0_* and *β_1_*, which is a fairly simple topological characteristic. The detailed description of the theory can be found in any good book on algebraic topology, e.g., Munkres [109], Hatcher [60], or Kaczynski, Mischaikow, and Mrozek [75]. At first sight, a formal theory might seem daunting, but the important fact is that a simple, almost trivial topological argument “no holes in trajectory spacetime” is sufficient to unambiguously determine sample clusters on different scales. The very nature of an event and a cluster of events is its “coherence” in space and time. Time comes with an order (consecutive) and space comes with a topology (vicinity, nearness).

**Figure 6:**
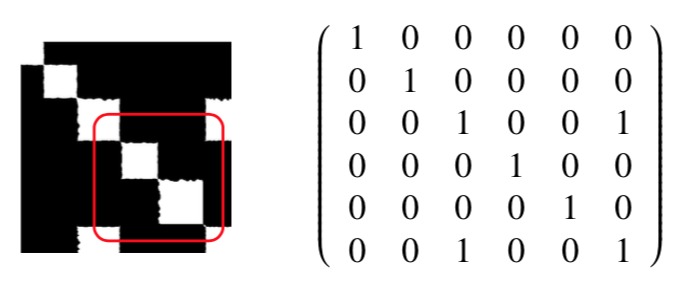
Matrix representation for scanpath

What we have obtained is the adjacency matrix A = [ai; j] of graph theory for our gaze trajectory. The side length of a square around the diagonal is proportional to the duration of fixation (the time scale is fixed by the sampling rate of the gaze tracker). The rectangles in the upper and lower triangular matrix represent a return (recurrence). The length of each block contains the time information, i.e., the duration of a cluster. Separating the blocks results in the sequence of fixations and their durations as well as the duration of intermediate gaps. Suppressing the time information in the matrix, i.e., shrinking the squares along the diagonal to one point entries, one arrives at the classical scanpath string representation of ABCDEC in the form of a matrix, see Figure 6.

The off-diagonal elements are the coupling, i.e., recurrence of the fixations. The same argument for the second level squares yields the dwells, i.e., one obtains (ABC)1(DE)2C1 (superscript numbers the dwell).

To summarize: for trajectory separation, three computational steps are needed. A distance representation for the gaze-trajectory in form of a time indexed matrix of all combinatorial 2-point distances is calculated. To separate the matrix into subparts a sliding threshold t is set, which is the sought diameter of a fixation. The threshold t is increased from 0 in steps and the number of connected parts, *β_0_*, and holes, *β_1_*, is traced. As soon as the square blocks along the diagonal form a simply connected area without holes, the minimum threshold tc for the segmentation into fixations has been found. Further raising the threshold yields the dwells.

### Abstract spacetime clustering

So far, the segmentation process for the gaze trajectory in screen space has been discussed, but the method can be made much more far-reaching. In order to do so, the meaning and interpretation of space will be generalized.

Up to now the concept of space has been the physical space and its Euclidean modeling, specifically its Euclidean metric. The crucial point is that the eyes, seen as a mechanical system, are moving in physical space, but the driving physiological and psychological processes are working in “physiological and psychological spaces”. An example of a physiological space is the color space and a much more complex space is the social space of humans when interacting, say, at a cocktail party. In this space the items or “points” are interlocutors, and the eyes are switching between these points with motivations such as signaling interest in the interlocutor’s small talk, which is a gesture of politeness, and does not have the primary goal of gathering visual information. Gathering information is looking at the face to feel out the mood, etc. What counts is not the physical distance between the interlocutors, but rather some sort of social communication-distance. Relevant are the “content” of the scene and the “strategy” of the observer while interacting, which in turn is reflected in the saccade-and-fixate pattern. Physical space-distance is not a restricted resource for the eyes. The eyes can move effortlessly from one point to each other point in physical space.

**Figure 7:**
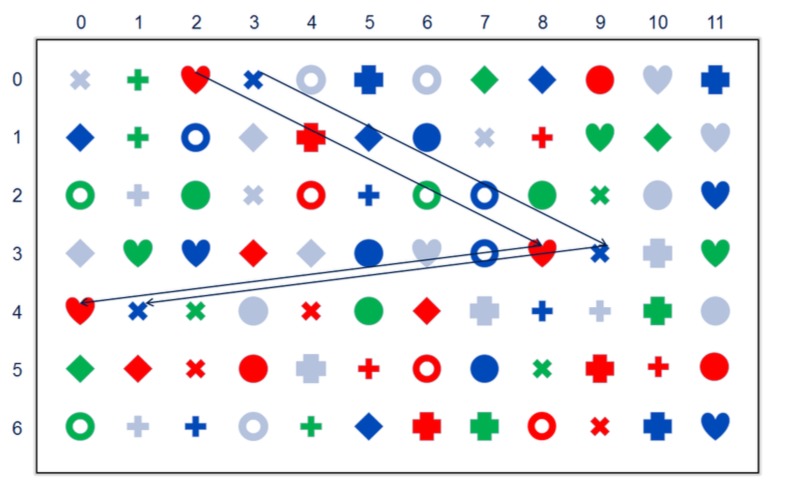
Search plus path

As an example for the approach try for yourself the following search paradigm, see Figure 7. In the collage of colored shapes all but two colored shapes occur three times, one colored shape occurs twice and another colored shape occurs four times: which two are they? Admittedly, searching for numerosity is hard! Nevertheless, numerosity is a good example for an abstract feature, not tied to a primary sensory input. You can track and visualize your own search strategy in the supplementary interactive document.

At the beginning many trajectories have fixations on a color. This derives from the fact that humans can identify color-blobs very easily in their view field. Thus, the first “search channel” is very often color.)^11^ The second channel is an easily detectable “geometry”. While the distinct color blobs are far apart in terms of geometric Euclidean distance they are near in colorspace, i.e., the red disk (0,9) is near, actually identical, in color to the red disks (5,3) and (5,11). The same holds true for the “geometry channel”, e.g., the motives with a circular boundary. It is likely that most subjects will start out with a random search strategy, which after a while will be abandoned in favor of a systematic, rowby- row, search strategy.

The qualitative approach to the geometric stimuli analysis is taken in “Gestaltpsychology". A more recent and formal approach to it is taken in structural information theory and algorithmic information theory, which can be made quantitative. Using specialized metrics differentiates the channels in the search strategy in a metric way and helps to classify viewers. It is helpful to change the terminology and to say that the eyes are moving in “feature space”. This space has different dimensions like color, shape, etc., which form subspaces. The feature space is a topological space. For ease of use it could be modeled as a metric space and the path is encoded in feature distance. Of course, the metric has to be adapted for special purposes. A simple example is the distance in color-space. Simple is certainly relative, taking into account the long way from first color theories of the 19^th^ century into the elaborated color spaces like the HUE space, used in printing and computer imaging. This development has by no means come to an end. A (much) more complex example is the distance in social interaction.

Nevertheless, the starting point is always the basic notion of a metrizable “neighborhood or nearness” relation in the form of a metric. The metric is the crucial starting point to emphasize different aspects in the trajectory. Let us start with the metric on a space X. The general mathematical notion of a metric is a function (Equation 2)

**Figure:**
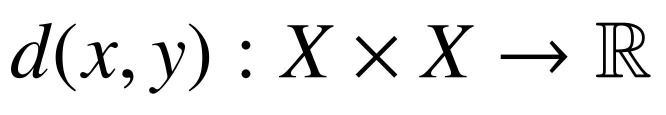


satisfying for all *x, y, z ϵ X* the conditions

Positiveness: d(x; y) ≥ 0 with equality only for x = y Symmetry: d(x; y) = d(y; x) Triangle inequality: d(x; y) ≤ d(x; z) + d(z; y)

This definition is only the bare skeleton of a metric. By itself it does not preassign any structure in the data, as is shown in the example:(Equation 3)

**Figure:**
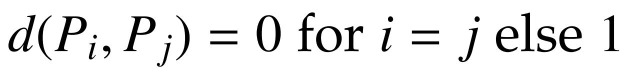


A more complex metric gives a much richer structure, emphasizing interesting aspects in the data. In RGB color space the distance between two colors C1(R;G; B) and C2(R;G; B) simply is: (Equation 4)

**Figure:**
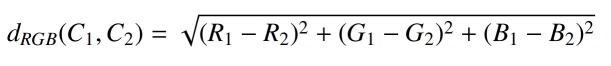


Adifferent example is reading. Here it would be appropriate to work within text space. For the understanding of reading patterns, not only the physical spacing of characters, but also the semantic distance is important. The semantic distance measures the difficulty of understanding words in a reading context. In the flow of reading, words can be physically close together, but if a word does not fit into the context or is not known to the reader, the reader will have difficulties in processing the word and a regression is most likely. Understanding a text requires coherence of word semantics as well as with the narrative in which they occur. The reader is traveling in general feature spaces and coherence is maintained or broken.

Along these lines more complex spaces can be constructed and analyzed. Clustering the data in feature space reveals directly the process related time ordering without intermediate separation of data into fixations, saccades, and then assigning areas of interest. The process pattern works directly on the items of interest. To cite Stark and Ellis [151] Sensory elements are semantic subfeatures of scenes or pictures being observed and motor elements are saccades that represent the syntactical structural or topological organization of the scene.

The ITop algorithm is essentially meant for stimulispace based analyses. The idea of directly connecting stimuli information and eye tracking data is also proposed in [3].

## Results for fixation identification

To show the algorithm’s potential for level one eyetracking data segmentation, a basic comparison with a state-of-the-art algorithm is given. An in-depth evaluation together with a MATLAB R reference implementation will be provided in a follow-up article.

**Figure 8:**
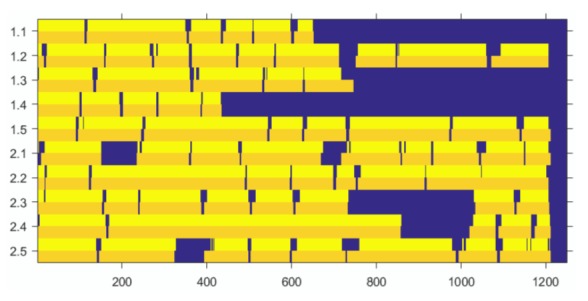
Performance of ITop and I2MC on ten datasets. The y-axis is in participant.trial, the x-axis is in samples. ITop fixation periods are in yellow and I2MC fixation periods are in orange. Dark blue is the gap between detected fixations or periods of data loss.

Current research has raised the awareness that algorithms commonly in use, especially when used “out of the box”, markedly differ in their results and an overall standard is lacking [3]. This situation escalates with each new algorithm proposed. The topological approach introduced herein is no exception. To make results comparable as much as possible a common reference set together with computed results, e.g., number and duration of events, event detected at samples, would be preferable. In a recent article, [63] introduced a new algorithm, identification by two-means clustering (I2MC), together with an open source reference implementation as well as ten datasets to show the performance of their approach. The I2MC algorithm is evaluated against seven state-of-the-art event detection algorithms and is reported to be the most robust to high noise and data loss levels, which makes it suitable for eye-tracking research with infants, school children, and certain patient groups. To ensure performance and comparability the identification by topological arguments (ITop) is checked against I2MC. The data are taken from www.github.com/royhessels/I2MC. The datasets comprise two participants, each participant having five trials, resulting in ten datasets overall. Both eyes are tracked. I2MC makes use of the data from both eyes for fixation detection, ITop classifies solely on the basis of the left eye data series. I2MC uses an interpolation algorithm for gap-filling. ITop works without gap filling. Figure 8 shows the classification results for the ten datasets under the ITop and I2MC algorithm.

**Figure 9:**
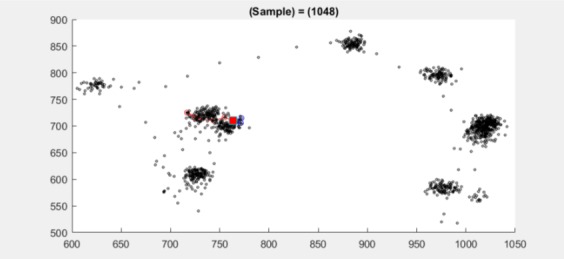
Scatter plot for dataset 2.2 at sample 1048 (red square at sample 1048) shows two clusters very close to each other

**Figure 10:**
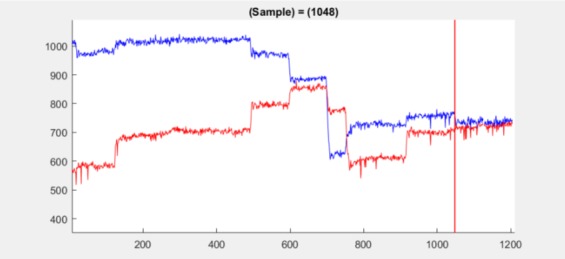
Position plot for dataset 2.2 at sample 1048 (red line at sample 1048) shows a small jump in the mean. The small jump is detected in spite of significant noise.

**Figure 11:**
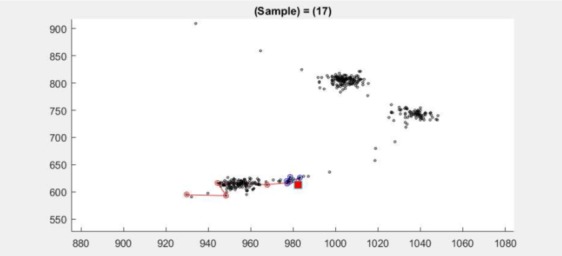
Scatter plot for dataset 2.3 at samples 17–19 (red square at sample 18) shows two clusters.

**Figure 12:**
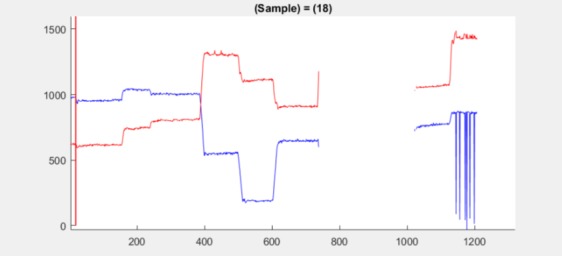
Position plot for dataset 2.3 at samples 17–19 (red line at sample 18) shows a small jump in the mean.

At some positions the ITop signal is splitted into two peaks, e.g., 1.3 (at samples 360–382 and 533–542) and 2.5 (at samples 1155–1165). This is no error, it is a finer view of the data. This is discussed in the following examples. The two approaches are in good agreement. Whenever I2MC detects a fixation ITop also does. ITop detects two additional fixations, one for 2.2 (at samples 1048–1049) and one for 2.3 (at samples 17–19). A closer look at the scatter plot as well as the position plot reveals two very close fixations, see (Figure 9, Figure 10) and (Figure 11, Figure 12).

**Figure 13:**
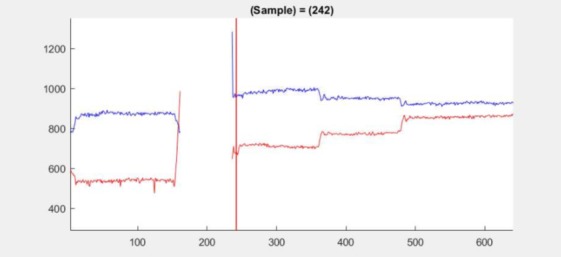
Position plot for dataset 2.1 at samples 242– 246 (red line at sample 242) shows a small jump in the mean after a period of data loss.

Although no data interpolation is done, ITop can identify a shift in the direct neighborhood of data loss. This is shown for 2.1 at samples 242–246, see Figure 13.

**Figure 14:**
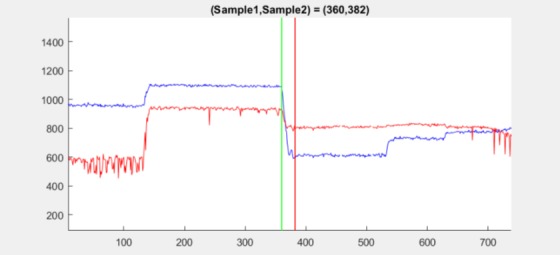
Position plot for dataset 1.3 between sample 360 (green line) and sample 382 (red line) showing a complex transit between two fixations.

At some positions the gap between fixations is split, e.g., for 1.3 at samples 360–382. This is a finer view of the data. As discussed, a saccade very often shows a complex stopping signal [65], post saccadic oscillations are a prominent example [116]. The term complex is meant in contrast to abrupt stopping. It does not necessarily mean a post-saccadic oscillation (PSO). A PSO is only an example for a named event with a more complicated “braking” pattern. This is reflected in the splitting of the signal. The position plot for 1.3 at samples 360–382 shows such a complex behavior, see Figure 14.

**Figure 15:**
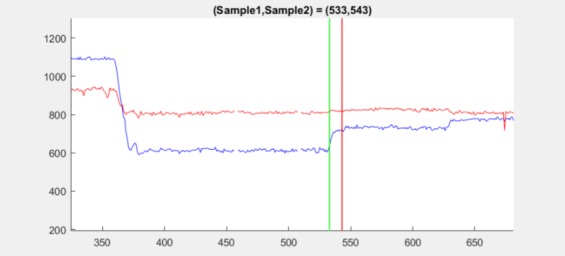
Position plot for dataset 1.3 between sample 533 (green line) and sample 543 (red line) showing a small jump in the mean of the y-position after stopping. The jump occurs at the red line.

The splitting according to braking can be much finer but is still detected by ITop. An example is 1.3 at samples 533–543. Here, a very small shift in the mean of the y-position signal occurs shortly after stopping, showing the high sensitivity of ITop, see Figure 15.

**Figure 16:**
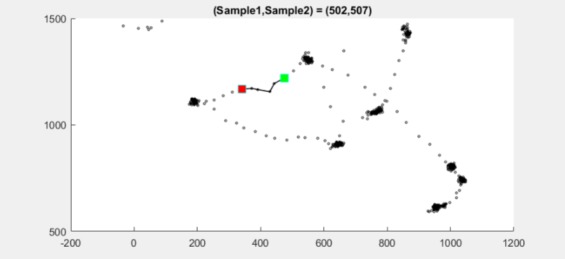
Scatter plot for dataset 2.3 between sample 502 (green square) and sample 507 (red square).

It must further be noted that the saccades according to ITop are longer (spatially wider) than under I2MC. As an example, dataset 2.3 at samples 499–515 is shown in detail. I2MC detects a gap between two fixations at samples 502–507, see Figure 16.

**Figure 17:**
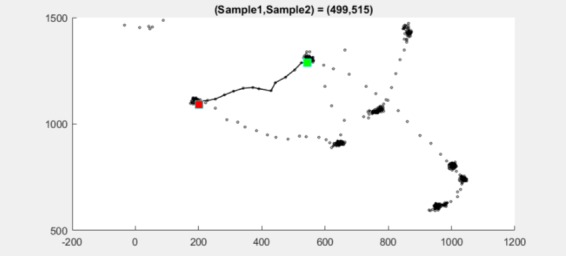
Scatter plot for dataset 2.3 between sample 499 (green square) and sample 515 (red square).

**Figure 18:**
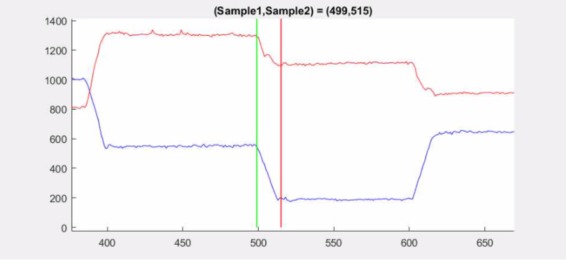
Position plot for dataset 2.3 between sample 499 (green line) and sample 515 (red line). A jag occurs at sample 504, potentially misleading algorithms.

**Figure 19:**
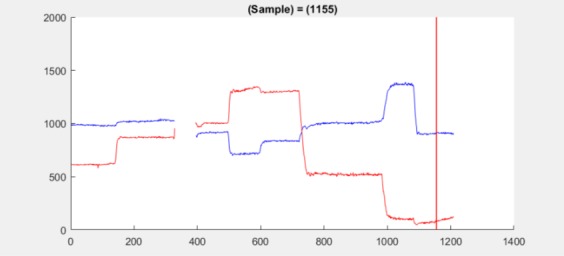
Position plot for dataset 2.5 shows a drift beginning at sample 1155 (red line).

ITop detects the gap at the same location at samples 499–515 and is therefore approximately twice as long, see Figure 17. The position plot shows a jag in the y-signal, which could potentially mislead an algorithm, see Figure 18. ITop also indicates other changes in the data series, like stationarity, e.g., the double peaked signal for dataset 2.5 at samples 1155–1165 indicates the onset of a drift in a fixation, see Figure 19.

Notwithstanding that I2MC and ITop are in good overall agreement they also show differences on a finer scale. If one takes into consideration the broad number of algorithms and different approaches for event detection it must be clear that the overall results can be markedly different. This can only be mitigated by defining events in an unambiguous and definite way and comparing algorithms on the basis of standard data on a sample by sample level.

## Discussion

A general overview of the algorithms currently in use for event detection in eye-tracking data is given, showing that there is no standard for event detection, even in the case of the most basic events such as fixations and saccades.

A topological approach to event detection in raw eye-tracking data is introduced, ITop. The detection is based on the topological abstraction of coherence in space and time of the sample points. The idea of trajectory spacetime coherence is given a precise meaning in topological terms, i.e., “no holes in trajectory spacetime”, a strikingly simple topological argument for the separation of the sample data. The topological argument is a kind of common rationale for most of the algorithms currently in use. The basis for the topological approach is the representation of raw eye-tracking data in the form of a time indexed matrix of combinatorial 2-point distances. This representation makes the coherence of sample data in space and time easyly accessible. The time ordered 2-point combinatorial distances representation makes the gaze trajectory independent of Euclidean motions, which is a desired property when comparing scanpaths, since distances are the invariants of Euclidean geometry.

For visualization, the matrix is displayed as a grayscale image to show the spatio-temporal ordering and coherence of the gaze-points in display space.

For the human visual system the interesting parts are easy to detect, e.g., fixations, dwells, etc. The visual assessment of spatio-temporal coherence is discussed and exemplified in the context of coarse-fine (globallocal) scanpath characteristics. It is argued that the visual assessment of the trajectory spacetime is helpful to identify general patterns in viewing behavior and to develop an intuitive understanding thereof.

To separate fixations and higher level clusters of fixations out of eye-tracking data, the common argument of spatio-temporal coherence, implicitly used in existing algorithms, is converted into an explicit topological argument, i.e., “no holes in trajectory spacetime”. The method encompasses the well known criteria which are partially expressed as thresholds for velocity, acceleration, amplitude, duration, etc. Tracking the number of connected parts and holes while varying the scale allows the partitioning of the distances matrix into the classical scanpath oculomotor events, i.e., segments of fixations and saccades. The segments are identified by their spatio-temporal coherence by means of simple homology, which is a classical tool of algebraic topology. For processing the data no preprocessing is needed, i.e., gap-filling, filtering, and smoothing, preserving the data “as is”. This approach makes it possible to identify the single events without any predefined parameters. A postprocessing of the found events, like merging of nearby fixations or the removal of physiologically implausible short fixations and saccades is not needed.

The topological segmentation is introduced in the familiar setting of Euclidean space and its well known metric. The advantage of this approach is that it can be easily expanded to general spaces like color spaces, shape spaces, etc., allowing the analysis of complex patterns in higher human activities. The ITop algorithm is essentially meant for stimuli-space based analysis.

In order to facilitate the intuitive understanding the article is accompanied by a supplementary interactive document.

ITop is considered as a fourth approach to eyetracking data in addition to the well known threshold based approaches and the newer probabilistic and machine learning methods. An expanded comparison, analysis, and classification of the ITop detection patterns together with an open source MATLAB R reference implementation will be provided in a further work.

## Acknowledgement

We thank the anonymous reviewers who provided helpful comments on earlier drafts of the manuscript and whose comments/suggestions helped to improve and clarify this manuscript. The provision of important references and preprints is also greatly appreciated.

## Footnotes

Realistically the model is a strong assumption (prior) and very often the hypothesized construct is driven by the original model.

(Mason, 1976[102]; Karsh and Breitenbach, 1983[77]; Widdel, 1984[179]; Scinto and Barnette, 1986[140]; Stampe, 1993[150]; Krauzlis and Miles, 1996[86]; Wyatt, 1998[183]; Salvucci and Goldberg, 2000[134]; Privitera and Stark, 2000[124]; Larsson, 2002[93]; Engbert and Kliegl, 2003[40]; Smeets and Hooge, 2003[146]; Santella and DeCarlo, 2004[135]; Engbert and Mergenthaler, 2006[41]; Urruty, Lew, and Ihadaddene, 2007[163]; Spakov and Miniotas, ˘ 2007[164]; Shic, Scassellati, and Chawarska, 2008 [145]; Camilli, Nacchia, Terenzi, and Nocera, 2008[25]; Kumar, Klingner, Puranik, Winograd, and Paepcke, 2008[87]; Munn, Stefano, and Pelz, 2008[110]; Blignaut, 2009[19]; Komogortsev, Jayarathna, Koh, and Gowda, 2009[82]; Nyström and Holmqvist, 2010[116]; Komogortsev, Gobert, Jayarathna, Koh, and Gowda, 2010[81]; Dorr, Jarodzka, and Barth, 2010[33]; van der Lans, Wedel, and Pieters, 2011[166]; Mould, Foster, Amano, and Oakley, 2012[108]; Komogortsev and Karpov, 2012/13[83]; Vidal, Bulling, and Gellersen, 2012[175]; Liston, Krukowski, and Stone, 2012[96]; Špakov, 2012 [176]; Valsecchi, Gegenfurtner, and Schütz, 2013[165])

Entia non sunt multiplicanda praeter necessitatem (Entities must not be multiplied beyond necessity). –John Punch–.

4In reading called gaze (Just and Carpenter, 1980 [73]) and in human factors called glance (Green, 2002 [53]). To avoid confusion with the standard meaning of gaze the term dwell is used (Holmqvist et al., 2011[64])

The term scanpath is somewhat vague and differs in its meaning and interpretation between different research areas and authors. Introduced in 1971 by Noton and Stark (Noton and Stark, 1971b, 1971a, 1971c[115, 116, 117]; Zangemeister, Stiehl, and Freksa, 1996[184]), it was a fairly abstract concept to describe a repetitive pattern of a single subject while viewing a static stimulus (Privitera, 2006[123]). Common terminology has been improved with works such as Holmqvist et al. (2011[64]), research networks such as COGAIN, or industry driven demands such as the ISO 15007 and SAE J2396 standards for in-vehicle visual demand measurements (Green, 2002 [53]).

wo-state Hidden Markov models (HMM) are intrinsically based on fitting individual data thus avoiding the problem of setting parameters explicitly (Salvucci and Goldberg, 2000 [134]; Rothkopf and Pelz, 2004[130]). A prerequisite is, however, to assume two states, i.e., saccade and fixation, limiting the classification.

E.g., a saccade epoch in the trajectory is in first approximation a straight line, which is a geometric shape feature (a closer look shows it is curved (Inhoff, Seymoura, Schad, and Greenberg, 2010[69]).

Here gaze is understood as the ray from the center of the entrance pupil and the point-of-regard, essentially the first part of the line-of-sight. For a detailed discussion of the related notions line-of-sight, pupillary axis, visual axis, etc. see (Bennett and Rabbetts, 2007[14]; Schwartz, 2013[139]).

Plotting a distance matrix is a technique used in different research areas and comes under different names, e.g., visual assessment of cluster tendency (VAT) (Havens, Bezdek, Keller, and Popescu, 2008 [61]; Bezdek and Hathaway, 2002[16]), or see, e.g., Junejo, Dexter, Laptev, and Pérez (2011[72]). In the context of dynamical systems it is called recurrence plot (Eckmann, Kamphorst, and Ruelle, 1987[38]). Recurrence analysis is a successful tool for describing complex dynamic systems, see, e.g., Marwan, Romano, Thiel, and Kurths (2007[101]). The reference also includes a simple statistical model for the movement of the eyes, i.e., the disrupted Brownian motion. Recurrence analysis is also known in eye movement research (Anderson, Bischof, Laidlaw, Risko, and Kingstone, 2013[2]; Farnand, Vaidyanathan, and Pelz, 2016[43]).

Anatomically a separate pathways for color can be distinguished (Schwartz, 2010[138]).
